# Cerebral Haemodynamics and Cognitive Impairment in Chronic Haemodialysis Patients: A Pilot Study

**DOI:** 10.3390/jcm14144890

**Published:** 2025-07-10

**Authors:** Giulia Belluardo, Dario Galeano, Concetto Sessa, Giuseppe Zelante, Walter Morale, Paola De Bartolo

**Affiliations:** 1 Department of Human Sciences, Guglielmo Marconi University, Via Plinio 44, 00193 Rome, Italy; p.debartolo@unimarconi.it; 2 Department of Nephrology and Dialysis, Maggiore Nino Baglieri Hospital, 97015 Modica, Italyconcetto.sessa@asp.rg.it (C.S.);; 3 Department of Neurology, Guzzardi Hospital, 97019 Vittoria, Italy; 4 Lab of Experimental Neurophysiology, IRCCS Santa Lucia Foundation, Via del Fosso di Fiorano 64, 00143 Rome, Italy

**Keywords:** haemodialysis, cognitive impairment, cerebrovascular disease, haemodynamic, carotid artery disease, neuropsychology, transcranial Doppler

## Abstract

**Background**: Patients with chronic kidney disease (CKD) have a substantially higher risk of developing cognitive impairment (CI) than the general population. Patients with CKD undergoing haemodialysis (HD) treatment also have an elevated risk of developing cerebrovascular and cardiovascular diseases. This study aims to investigate the relationship between the cognitive performance of haemodialysis patients and cerebral and carotid haemodynamic indices. **Methods**: This study was a non-interventional observational study; the sample consisted of 32 patients (age 65 ± 12 years) undergoing chronic HD treatment. The patients underwent neuropsychological and haemodynamic instrumental investigations, including Supra-Aortic Trunk Echodoppler (SAT) and Transcranial Doppler (TCD). **Results**: Patients were 17% deficient at Montreal Cognitive Assessment (MoCA), 45% deficient at Frontal Assessment Battery (FAB), 55% deficient at Trail-Making Test-A (TMT-A) and 65% deficient at TMT-B. The TCD investigation detected a decrease in flow (MFV) and an increase in Breath Hold Index (BHI) predominantly in the right cerebral arterial district. The SAT investigation revealed an altered IMT, plaques and the presence of severe carotid stenosis. A strong association between cerebral and carotid indices and cognitive scores was also observed. Correlation analyses reported statistically significant correlations between TMT-A and TMT-B and cerebral flow indices. **Conclusions**: Among haemodialysis patients, there is a high percentage of cognitive impairment associated and correlated with alterations in cerebral and carotid haemodynamics. Cerebral haemodynamics are a factor to be taken into consideration as a possible pathological mechanism underlying cognitive impairment in haemodialysis.

## 1. Introduction

Chronic kidney disease (CKD) has a rapidly increasing worldwide prevalence and is recognised as a growing public health problem. Patients with CKD have a substantially higher risk of developing cognitive impairment (CI) than the general population [1]. The mechanisms that may contribute to CI are multiple, some attributable to a susceptibility that the kidneys and brain possess to traditional vascular risk factors, such as diabetes mellitus, atrial fibrillation, dyslipidaemia and hypertension, and others attributable to non-traditional CKD-related risk factors, such as uraemia, oxidative stress, chronic inflammation, hyperuricemia and electrolyte imbalances [2]. Among the mechanisms that may lead to CKD-related risk factors are cerebrovascular and cardiovascular alterations. CKD is a known risk factor for the development of cerebrovascular and cardiovascular diseases. A reduced glomerular filtration rate (GFR) increases the risk of experiencing stroke events by approximately 40% and hurts cerebral autoregulation, leading to a remodelling of the cerebral vasculature and a reduction in cerebral blood flow (CBF) [2,3,4,5,6]. From a cardiovascular point of view, renal function is also associated with the development of coronary artery disease (CAD), with GFR being a strong predictor of carotid intima-media thickness (IMT), progression of atherosclerosis and increased fatal vascular events. Patients with CKD also have a high risk of developing carotid calcifications and unstable plaques that have a high probability of rupturing and causing cerebrovascular embolic events [2,7,8]. The risk of cerebrovascular and cardiovascular adverse events increases when patients with CKD, who have arrived at the end-stage of the disease (ESRD), are initialised on haemodialysis (HD) treatment. Because HD uses an extracorporeal mechanism of fluid removal and blood purification, patients often experience haemodynamic changes during sessions. The kidneys and brain are particularly susceptible to vascular damage, and microvascular regulation in both organs is anatomically and functionally similar: both are high-flow organs and rely on local autoregulation. Haemodynamic alterations and changes in blood composition following HD lead to increased vascular permeability, endothelial dysfunction and cerebral pathologies such as stroke, with which haemodynamic instability is strongly correlated [9,10,11].

According to [9,10], HD patients have an 8–10 times higher incidence of stroke than the general population, with a higher prevalence of haemorrhagic stroke, which accounts for 20% of all stroke events [12,13]. Specifically, the initiation of chronic HD treatment seems to be associated with an increased risk of stroke; in particular, rates of stroke events increase about 3 months before the start of HD and peak during the first 30 days of HD [14]. From this evidence, it has been hypothesised that a repetitive decline in CBF resulting from multiple intra-week HD sessions may lead to the formation of ischemic brain lesions, and that these, in turn, may be accompanied by cognitive decline [15]. The incidence of CAD also increases after the start of HD treatment, with the increase being more pronounced as the HD treatment time lengthens [16]. Growing evidence links CAD to the development of CI, identifying carotid artery disease as a predictor of cognitive function [17]. It is clear, therefore, that HD has an exacerbating effect on cerebrovascular and cardiovascular issues and that the haemodynamic instability caused by HD is a relevant factor in the development of CI.

One of the less-invasive instruments used for assessing cerebral perfusion is the Transcranial Doppler (TCD), which has the advantage of being able to be performed at the patient’s bedside, allowing the flow velocity of intracranial arteries to be measured in real-time. A reduction in mean cerebral flow velocity (MFV) associated with an increase in ultrafiltration volume (UF) has been detected in the HD population by TCD [18]. Most studies on HD patients have investigated haemodynamic changes during sessions and have compared CBF immediately before and immediately after the HD session, assessing the impact of these changes on cognition. However, there are no studies that have investigated cerebral perfusion and cerebral autoregulation in HD patients on non-dialysis days, when patients are not burdened by the accumulation of waste products or influenced by the purifying and haemodynamically stressful effect of HD.

Similarly, Supra-Aortic Trunk (SAT) Ultrasound is a non-invasive test typically used to detect the typical signs of CAD, which can provide information on the integrity of the carotid arteries [19]. After these assumptions about cerebrovascular and cardiovascular risks in the HD population, we hypothesised that decreased CFB and the presence of CAD may be significantly associated with the CI observed in the HD population. Given the lack of studies on cognitive impairment in HD employing both TDC and SAT during a non-dialysis day in the literature, this pilot study aims to utilise both instruments and contextually investigate both CBF and signs of CAD in HD and their relationship with cognitive performance.

## 2. Materials and Methods

### 2.1. Subjects and Study Design

An observational, open-label prospective pilot study was performed. The sample size required for the correlation models to have sufficient statistical power was calculated using the Events Per Variable (EPV) method [20]. The EPV method calculates the ratio between the sample size and the number of independent variables that can be included in a correlation model while maintaining its statistical power. Therefore, calculating an EPV is given by the following: EPV = 1/15 = 0.067.


**EPV (coeff = 0.067)**

**Sample Size**

**Model Variables**
0.067 x100.67 => approximated to10.067 x20
**1.34 => approximated to 1**
0.067 x302.01 => approximated to 2
**0.067 x**

**32**

**2.144 => approximated to 2**
0.067 x9060.067 x 10070.067 x150100.067 x200130.067 x300Max 20 var

For a correlation with one independent variable per model, the minimum sample size is 20 patients. A total of 32 patients (20 males and 12 females, 65 ± 12 of age) were recruited from three HD centres: the Nephrology and Dialysis Department of Maggiore—“Nino Baglieri” Hospital in Modica, the Dialysis Service of Maria Paternò Arezzo Hospital in Ragusa and the Decentralised Care Dialysis centre of Busacca Hospital in Scicli. After reading the information sheet and obtaining informed consent in accordance with the guidelines proposed by the Declaration of Helsinki of the World Medical Association, adult patients with End-Stage Renal Disease (ESRD) undergoing chronic HD for at least 3 months, aged over 18 years and of both sexes, were enrolled. All patients included in the study were treated with conventional thrice-weekly HD sessions, each lasting approximately four hours. Dialysis was performed either using the Fresenius 5008 system (Fresenius Medical Care, Fresenius SE & Co. KGaA, Bad Homburg vor der Höhe, Germany) or the AFB (Automated Functional Balance) system by Baxter. Each centre applied a consistent dialysis modality for its patients throughout the study. Patients treated with the Fresenius 5008 system received high-flux bicarbonate dialysis with Helixone^®^ synthetic membranes, composed of polyethersulfone, with surface areas ranging from 1.8 to 2.2 m^2^ and ultrafiltration coefficients between 45 and 70 mL/h/mmHg. The dialysate was ultrapure and bicarbonate-based, with a sodium concentration between 138 and 142 mmol/L, bicarbonate ranging from 32 to 35 mmol/L, potassium individualised between 1.5 and 3.0 mmol/L and calcium set between 1.25 and 1.50 mmol/L. Water treatment was ensured by a double reverse osmosis system in accordance with international standards. Conversely, patients undergoing treatment with the Baxter system received dialysis based on the AFB protocol, which maintains a constant potassium concentration and modulates sodium and bicarbonate delivery according to patient-specific needs. These sessions were carried out using AN69^®^ membranes, composed of acrylonitrile and sodium methallyl sulfonate copolymer. Infusion flows were automatically calculated according to standard modelling conditions, assuming a haematocrit of 30%, a dry weight of 60 kg, an ultrafiltration rate of 1 kg/h and a target increase in bicarbonataemia from 18 to 28 mEq/L. These values were used by the system to dynamically adjust convective and diffusive exchange during the session. Dialysis efficiency was monitored using single-pool Kt/V, with an average of 1.4 ± 0.2. Blood flow rates ranged from 300 to 350 mL/min. Vascular access consisted of either arteriovenous fistulae, tunnelled central venous catheters, or arteriovenous grafts, depending on the individual clinical condition.

On the contrary, patients presenting the following issues were excluded from the study:

1. Diagnosis or evidence in neuroradiological imaging of established cerebrovascular disease prior to HD (ischemic and/or haemorrhagic stroke);
2. Evidence of small vessel disease prior to HD;
3. Previous diagnosis of a severe psychiatric disorder (e.g., schizophrenia, major depression, bipolar disorder);
4. Previous neurosurgical interventions;
5. Inability to perform apnoea testing because of respiratory failure or any other reason;
6. Unable to complete neuropsychological tests;
7. Previous diagnosis of atheroembolic disease;
8. Previous endarterectomy interventions and/or carotid stenting.

Before undergoing the research phase, all patients were informed about the overall purpose of the study and the methods, procedures and timings used. After the recruitment phase, the following variables were collected, provided that they were available in patients’ medical records:

1. Demographic data (age, sex, weight, height, Body Mass Index, Body Surface Area);
2. Relevant medical history;
3. Comorbidity (hypertension, diabetes, dyslipidaemia, coronary artery disease, atrial fibrillation, Peripheral Arterial Obstructive Disease, Systemic Lupus Erythematosus, Fabry disease);
4. Dialytic data (HD age, filter type, haemodialytic mode, filter surface, mean intradialytic pressure, vascular access, average Kt/V, sodium and bicarbonate conductivity);
5. Secondary complications of ESRD (anaemia, inflammation, hyperparathyroidism, dyslipidaemia).

The enrolled patients were assessed by means of neuropsychological tests and by TCD and SAT, during a day off from HD. The neuropsychological assessments were conducted before the TCD and SAT were taken, in order to avoid impairing cognitive performance due to possible fatigue from the instrumental examinations. The duration of the cognitive assessment was about thirty minutes. The duration of the TCD was about ten minutes, and the duration of the SAT was about thirty minutes.

### 2.2. Instruments

a. *Neuropsychological assessment*
  To evaluate the cognitive functions of patients undergoing HD, specific tests were selected to provide a comprehensive cognitive assessment.
  - **Montreal Cognitive Assessment (MoCa)**
    It is a short first-level evaluation battery widely used in the neuropsychological domain. The battery includes a series of items that examine visual–spatial and executive functions (Trail-Making Test, Clock-Drawing Test, Cube Imitation Test), naming, attention (in particular, selective attention, sustained attention and serial calculation or “series of 7”), language (through repetition and fluency items), abstraction, delayed recall (of five words) and, finally, orientation (day, month, year, place, plan). The test administration time is 10–15 minutes and provides a draft of a possible cognitive impairment. The maximum battery score is 30 and the raw score obtained by administration must be adjusted by means of tables of correction in terms of age and education level. Santangelo’s standardisation was used in this study [21]. The cut-off of MoCa with Santangelo’s standardisation is equal to 15.5. A score of <15.5 is a wake-up call for cognitive impairment, a score between 15.5 and 17.54 suggests borderline performance, while a score of >17.54 is associated with normal cognitive functioning.
  - **Frontal Assessment Battery (FAB)**
    It is a useful battery to evaluate the functioning of one’s executive functions, a set of faculties generally located in the frontal areas of the brain that underlie different processes involved in flexibility, working memory, focused attention, motor planning and the inhibition of response. This battery consists of six cognitive tests and each of them has a score ranging from 0 to 3; the overall range is 0–18 and the administration time does not exceed 10 minutes on average. Specifically, it includes the following tests: similarities, lexical fluency, motor series with the dominant hand (Luria’s Sequence or “fist-edge-palm” test), response to conflicting instructions, go-no-go and prehension behaviour. For the present study, the Italian Apollonio standardisation was applied, as it is suitable for a population with a minimum age of 20 and a maximum age of 95, and years of education ranging from 0 to 13 [22]. The cut-off was identified in the score of 13.50. Afterwards, the raw score had to be corrected by age and education level and converted into equivalent scores.
  - **Trail-Making Test A and B (TMT-A/B)**
    They are two tests which assess spatial planning ability in visual–motor tasks and attention skills. Patients are invited to perform the test as quickly as possible. The final score depends on the seconds used to perform the test. For this study, the ENB-2 standardisation (Short Neuropsychological Examination 2) developed by Mondini—which can be administered to patients with a minimum age of 15 and a maximum age of 96 and has a correction based on age and education level (below or above 8 years)—was used [23].
b. *Vascular assessment*
  To evaluate the vascular features of patients undergoing HD, the following instruments were used:
  - **Transcranial Doppler**
    Transcranial Doppler (TCD) conducted with a low-frequency probe (≤2 MHz) allows us to monitor CBF velocity and vessel pulsatility over relatively short to extended periods and to assess cerebral autoregulation. The acoustic window used in this study was the transtemporal window, located above the zygomatic arch, at a midpoint between the canthus of the eye and the auricle. The cerebral artery taken into consideration was the middle cerebral artery (MCA). The indices collected were the following: Peak Systolic Velocity (PSV), End-diastolic Velocity (EDV), Mean Flow Velocity (MFV), Gosling’s Pulsatility Index (PI), Pourcelot’s Resistance Index (RI) and the Breath Hold Index (BHI). MFV can be influenced by factors such as age, sex, mean arterial pressure and haematocrit. Normal values for the MCA for adults are 55 ± 12 cm/s. An increased value may be an index of stenosis, vasospasm or hyperdynamic flow, while a reduced value may indicate hypotension, decreased CFB and intracranial pressure (ICP) or brain death. Gosling’s Pulsatility Index (PI) provides information on downstream cerebral vascular resistance and is calculated as follows: (PSV-EDV)/MFV. Standard values are within a range of 0.5–1.19. Lower values may correspond to stenosis, proximal occlusions, arteriovenous malformation of the brain; higher values may indicate distal occlusion or stenosis and intracranial hypertension. The RI corresponds to (PSV-EDV) /PSV. Values greater than 0.8 indicate increased downstream resistance. The BHI, instead, provides an estimate of the ability to autoregulate brain pressure following a few seconds of apnoea (25/30 s) after which, in a non-pathological situation, there should be an increase in the velocity of flows and therefore of the MFV [24]. The calculation of the BHI is as follows: (MFV test—MFV baseline) /MFV baseline X (100/s of breath holding) [25]. In this study, a Chison XBit 70 ultrasound machine—with Chison sector probe (≤2 MHz)—was used to perform the TCD. The soundproofed cerebral artery was the MCA through the transtemporal window.
  - **Supra-Aortic Arterial Trunk Echocolourdoppler**
    The Supra-Aortic Arterial Trunk Ultrasound (SAT) is a diagnostic test usually used for the detection of signals typical of atherosclerotic disease of the extracranial carotid district. High-frequency probes (from 7.5 to 12 MHz) are used for this examination. The examination consists of two phases: the former is in B-mode and Color-flow on a transversal and longitudinal plan; the latter is by Doppler pulsed on the longitudinal plan. During the former phase in B-mode and Color-flow, the percentage of possible stenosis, presence of plaques and the intima-media thickness (IMT) are detected. Standard values of IMT are influenced by age and sex. A value below 0.9 is considered standard, while higher values are markers of atherosclerosis. With regard to the percentage of stenosis, it was calculated using the NASCET method. The plaques—in addition to being displayed in B-mode and analysed through the Grey Scale—can be examined by the Color-Doppler to evaluate the haemodynamic framework that accompanies them and to mainly verify the presence of aliasing, which is flow acceleration. A third-phase examination refers to the spectral analysis from which essential parameters are obtained to evaluate the severity of any haemodynamic stenosis. The systolic–diastolic velocity (PSV/EDV) increases as the degree of stenosis increases, since they are usually related. If stenosis exceeds 90% of the diameter reduction, the flow velocity decreases. In this study, a Chison X Bit 70 ultrasound machine, in B-mode and in Color-Doppler mode, was used. The following variables were collected for the Common Carotid Artery (CCA) and the Internal Carotid Artery (ICA), both on the right and on the left: IMT, number of plaques, EDV, PSV and percentage of stenosis (NASCET method) [26].

### 2.3. Data Collection and Statistical Analysis

Data were collected anonymously and stored on the experimenter’s computer, which was protected by passwords and antivirus software. The statistical analysis was conducted through the Jamovi software v 2.6.44. Data were processed to obtain descriptive statistics of the sample characteristics, including average values, standard deviations and percentages, based on the data obtained from the TCD and SAT, as well as the cognitive data collected from the neuropsychological questionnaires administered. After verifying the normal distribution of all variables through the Shapiro–Wilk test and T-Test, Spearman’s rho and linear regression were used to investigate possible statistically significant associations and correlations between brain/carotid data and cognitive data and to identify potential predictors of cognitive decline in HD patients. Bonferroni correction was applied for multiple comparisons.

## 3. Results

Data were obtained from 32 recruited patients (20 male and 12 female). Height and weight data were detected to calculate BMI and BSA. The average BMI of the population was 25, while the average BSA of the sample was 2. BMI and BSA were calculated with the body weight detected at the end of HD treatment (Table 1).

Data on the presence and prevalence of traditional and non-traditional risk factors in our population were collected using descriptive statistics, including percentages, average values and standard deviations. The results are all presented below in Table 2.

Furthermore, HD data from each patient were also collected through their medical records. As summarised in Table 3, specific characteristics of the HD treatment administered to each patient were identified.

### 3.1. Cognitive Results

The following tables show the results obtained through the administration of cognitive tests to patients undergoing HD included in the study. In order they are the Montreal Cognitive Assessment (MoCa), Frontal Assessment Battery (FAB) and Trail-Making Test (TMT-A/B) (Table 3).

All patients agreed to be tested (*n* = 32 tests). At MoCa, the lowest scores obtained by patients are found in the visual–spatial/executive domain, with an average score of 2.4 (out of an overall score of 5), in the attention domain with an average score of 4.4 (out of an overall score of 6) and in the delayed recall domain with an average score of 1.8 (out of an overall score of 5). The average of the corrected overall score of the sample is set at 19.3. A total of 17% of the sample reported a deficit score, 24% a borderline score and 52.6% provided standard cognitive performances. The corrected global average score of patients at the FAB stops at a value of 13.2 and therefore, on the equivalent score scale, it stands out as being in the deficit area. In fact, 45% of patients showed deficit performances, 17% of patients showed borderline performances, 41% of scores fell in the lower average area and 3% of scores fell in the upper average area. The most affected cognitive domains are motor planning (1.9 out of a total score of 3), go-no-go (1.8 out of a total score of 3) and phonemic fluency (2.2 out of a total score of 3). To complete the TMT-A, patients took an average of 110 sec and 55.1% of them had a below-standard cognitive performance. For the TMT-B, patients took an average of 243 sec and 65.5% of them had a below-standard cognitive performance. The cut-off is customised according to age and education.

### 3.2. Transcranial Doppler Results

Table 4 summarises the results of the indices chosen for the study of the MCA in HD patients: PSV, EDV, MFV (BHI baseline/test), PI, RI and BHI. Out of a total of 32 patients, 60% (19 patients) performed the test. Out of these 19 patients, 5 did not present the acoustic temporal window on the left and 2 did not present it on the right. Below are the data collected in the baseline condition and via the Breath Hold test. Standard reference values are also included in the table to facilitate comparison between the sample values and those of the general population. The flow velocities (PSV/EDV) on both left and right seem to fall on average within the reference range. The MFV on the left is at the standard’s limit (45.4 cm/s), while on the right it is definitely below-standard (41.6 cm/s). Bilaterally, RI are within standard ranges, as well as the pulsatility indices. At the BHI, the MFV is bilaterally increased. The BHI is standard on the left and increased on the right (1.75) (Table 4).

#### TCD and Cognitive Results

The indices recorded on the right and left using the TCD were matched to the overall corrected results of the MoCa, FAB and TMT (A-B) and the T-Test was applied to verify a possible association between the two types of variables. The significance level when Bonferroni correction is applied is *p* < 0.012.

**Figure 1:**
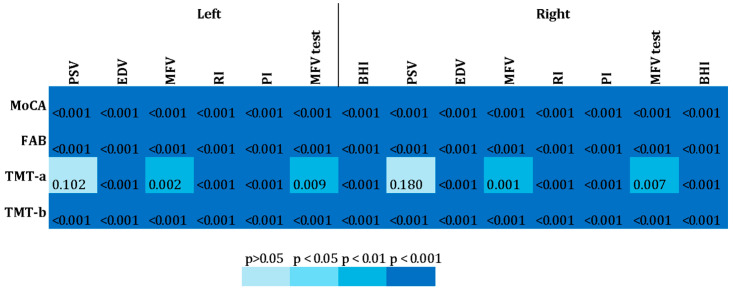
Heatmap of TCD and cognitive data. The figure shows a heatmap of the levels of association among various cognitive test and SAT data of (a) the left cerebral district and (b) the right cerebral district.

Significant and strong associations were found between all cerebral and cognitive variables examined, except for the TMT-A and the PSV of the left and right cerebral district (Figure 1). For further details, see Supplementary Table S1.

**Figure 2:**
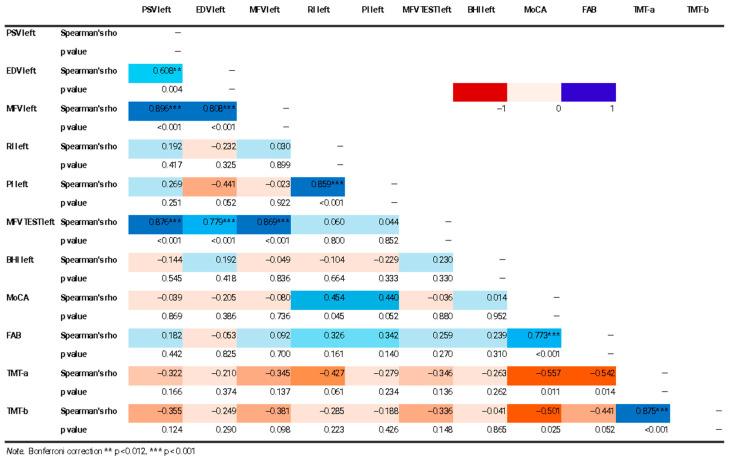
Correlation matrix heatmap of TCD and cognitive data. The figure shows a correlation matrix heatmap of left TCD and cognitive data. The Spearman correlation coefficient value (Rho) and statistical significance level (*p* value) of the results are shown.

**Figure 3:**
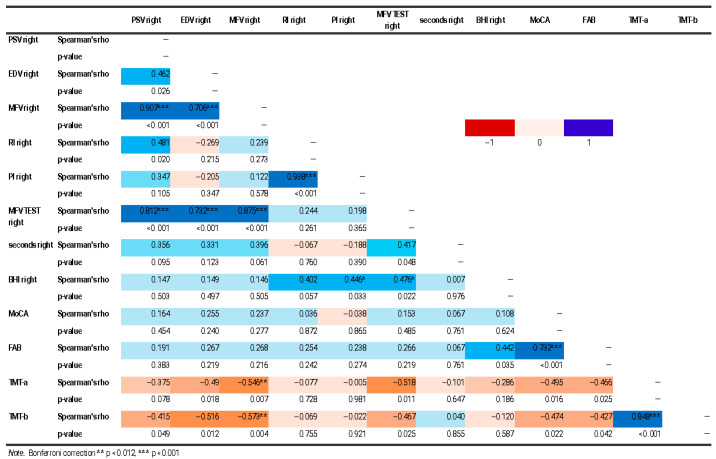
Correlation matrix heatmap of right TCD and cognitive data. The figure shows a correlation matrix heatmap of right TCD and cognitive data. The Spearman correlation coefficient value (Rho) and statistical significance level (*p* value) of the results are shown. Statistical significance was marked (* *p* < 0.05, ** *p* < 0.012, *** *p* < 0.001).

**Figure 4:**
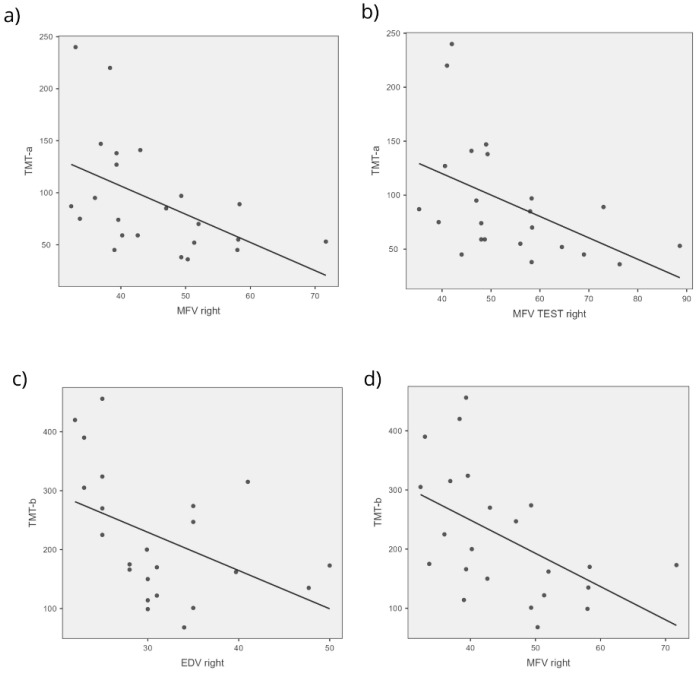
TCD and cognitive relationship. The figure shows scatterplots of the statistically significant relationships between the following: (**a**) Trail-Making Test-a and the MFV of the right district, (**b**) Trail-Making Test-a and the MFV test of the right district, (**c**) Trail-Making Test-b and the EDV of the right district, (**d**) Trail-Making Test-b and the MFV of the right district.

Through the application of Spearman’s correlation, some statistically significant correlations were found between brain indices and cognitive scores. Negative correlations were found between TMT-a and many flow indices on the right (*p* 0.018; MFV Rho −0.546, *p* 0.007; MFV TEST Rho −0.518, *p* 0.011) and the TMT-b and two flow indices on the right (EDV Rho −0.516, *p* 0.012; MFV Rho −0.573, *p* 0.004) (Figure 2, Figure 3 and Figure 4).

### 3.3. Supra-Aortic Arterial Trunk Echocolourdoppler

Table 5 summarises the results obtained by Supra-Aortic Arterial Trunk Ultrasound. The test was performed on a total of 23 patients in B-Mode and Doppler. The average values (IMT, plaque number, flow velocities) and the percentages (stenosis) of the obtained data were evaluated at a descriptive level. All data refer to the ranges of standard values within which they should be set for a quick comparison between the values of our sample and the reference ones. The IMT on the right seems to be at the standard’s limits, while at the level of the left carotid region it exceeds the limits, with IMT(sx) of 1.7 mm. There are one or more carotid plaques on both sides of the sample. The evaluation of stenosis in B-mode on the left almost reaches 50%. In Doppler mode on the right, 23% have a degree of severe stenosis (70%), 61% have a moderate degree of stenosis (50–69%) and the remaining 15% have mild stenosis (<50%); on the left, instead, there does not seem to be severe stenosis on average; 92% have moderate stenosis (50–69) and 7% mild stenosis (<50%).

#### SAT and Cognitive Results

The carotid indices recorded on the right and left by the Supra-Aortic Arterial Trunk Echocolourdoppler were matched with the overall corrected results of the MoCA, the FAB and the TMT-A/B. T-Test and Spearman Rho were applied to verify a possible significant association and correlation between the two types of variables.

**Figure 5:**
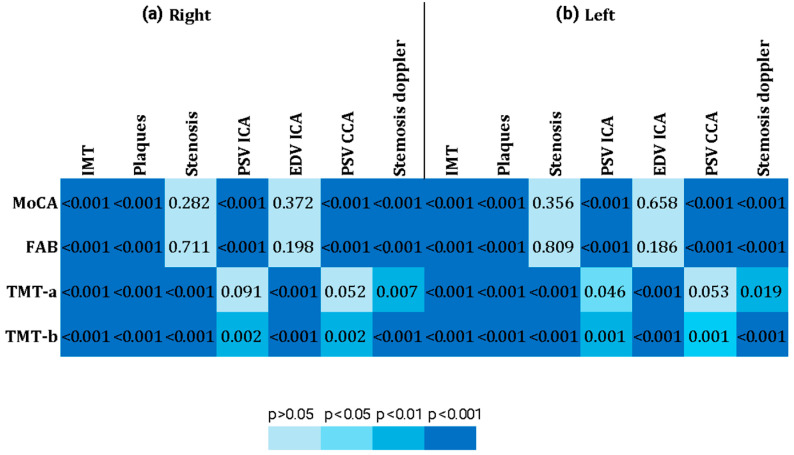
Heatmap of SAT and cognitive data. The figure shows a heatmap of the levels of association among various cognitive tests and the SAT data of (**a**) the right carotid district and (**b**) the left carotid district.

Significant and strong associations were found between all variables examined, except for MoCA and FAB, with the degree of stenosis (B-mode) and the EDV of the right and left carotid district, and, except for TMT-a, with the PSV ICA and PSV CCA of the right and left carotid district and with stenosis (Doppler mode) of the left district (Figure 5). For further details, see supplementary Table S2.

**Figure 6:**
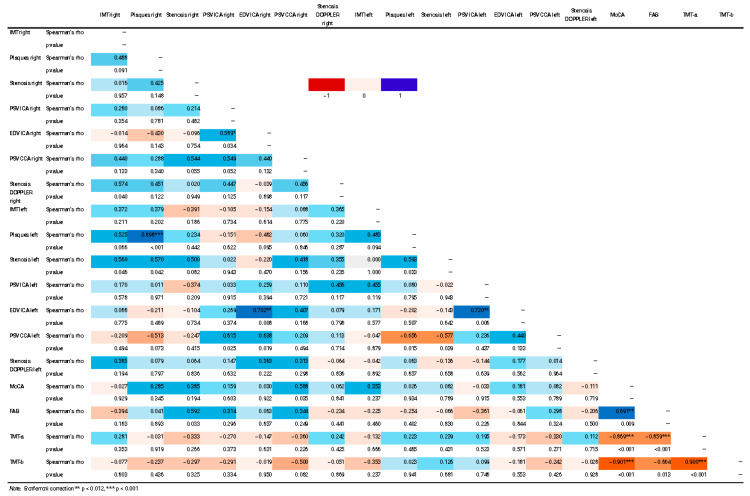
Correlation matrix heatmap of SAT and cognitive data. The figure shows Spearman’s correlations between SAT and cognitive scores. The Spearman correlation coefficient value (Rho) and statistical significance level (*p* value) of the results are shown.

No significant correlations (Bonferroni *p* < 0.012) were obtained between cognitive data and SAT data (Figure 6). Linear regression analysis also did not reveal any statistically significant predictors (*p* < 0.12); however, some regression models explain the variability of the dependent variable with a good percentage. Among these, the TMT/a- TCD RIGHT model explains 60% of the variability (R^2^ 0.602); the MoCA-SAT RIGHT explains 67% of the variability (R^2^ 0.673); the FAB-SAT RIGHT model explains 87% of the variability (R^2^ 0.875); the TMT/a—SAT RIGHT model explains 83% of the variability (R^2^ 0.836) and the TMT/a—SAT LEFT and TMT/b -SAT LEFT models explain 66% of the variability (R^2^ 0.660; R^2^ 0.661). For further details, see Supplementary Tables S3 and S4.

## 4. Discussion

In agreement with the literature, this study reveals a considerable percentage of cognitive impairment in HD patients, primarily affecting executive and visuospatial functions. Of the tests used in this study, the one that showed the greatest sensitivity in detecting this executive deficit was the FAB, although an executive component is also present in the MoCA. The domains most impaired in HD patients appear to be planning, inhibition, memory, particularly delayed recall, visual attention and visuospatial processing speed. Executive dysfunction is a hallmark of CI in HD patients; several studies have already reported a predominance of this type of deficit within this population, and it seems that it may be a symptom of dementia of a vascular nature [27,28]. On the brain side, the TCD examination showed a bilateral decrease in MFV and an increase in vasoreactivity, especially in the right arterial region. In our study, all indices of cerebral flow and vasoreactivity are significantly associated with the cognitive performance of HD patients, except for the PSV, with the performance obtained by MoCA. Cerebral haemodynamic impairment has been investigated over the last decade in various populations with cerebrovascular disease utilising neuroimaging and ultrasound techniques, and is independently associated with cognitive decline [29]. Our study also confirms this association in the HD population. A key finding of this study is the correlation between several cerebrovascular indices and cognitive performance. In particular, negative correlations were found between brain flow indices and cognitive performance, primarily related to visual–spatial processing speed and visual attention. In particular, the more brain flow indices decrease, the worse the cognitive performance of HD patients. Importantly, impaired CBF is generally associated with a faster rate of cognitive decline compared to normal cognitive ageing [30]. Our study, therefore, supports the role of hypoperfusion in the pathological progression of CI. Strong associations were also found between carotid indices and cognitive performance, indicating that the integrity of the carotid district, not just the brain, plays a role in the onset of CI in the HD population. Although there are many factors that contribute to the development of dementia, the literature, on the other hand, identifies CAD as one of the factors contributing to the development of cognitive dysfunction through carotid stiffness, increased carotid IMT, carotid stenosis and the risky presence of carotid plaques [31]. Our investigations of the HD population reveal some of these alterations, such as the thickening of the middle carotid intima, the presence of carotid plaques and a high prevalence of moderate carotid stenosis. Our regression analyses did not identify any statistically significant predictors of cognitive decline in this population; however, the cerebral models, and especially the carotid models, can explain the variance in cognitive performance with percentages ranging from 60 to 87%. In general, although the etiopathogenetic mechanisms underlying CI in patients with CKD have not yet been clearly established in the literature and can be attributed to a wide variety of factors, cerebral haemodynamics seem to be a determining factor that is specific to the segment of patients with CKD who undergo HD treatment. A recent study found that patients with HD have reduced cerebrovascular reactivity (CVR) compared to those with CKD and healthy subjects. Since CVR is established as a non-invasive marker of cerebrovascular health, its impairment could predispose HD patients to cerebrovascular damage and cognitive decline [32]. Continuous monitoring of the indices of cardiovascular and cerebrovascular integrity thus appears to be an indispensable strategy to adopt for the adequate clinical management of HD patients; from an experimental point of view, targeting attention to the cardiovascular and cerebrovascular aspect of these patients could lead to a greater understanding of the mechanisms underlying cognitive decline in HD and lead to the creation of new effective prevention strategies. One of the major limitations of our study is the sample size; our sample consists of only a few patients, which makes it difficult to generalise the results obtained to the entire HD population. Furthermore, the number of women is smaller than that of the number of men examined, which could reflect gender differences related to renal failure, which seems to affect the male sex more [33]. The limited sample size is also due to certain exclusion criteria, such as the presence of cerebrovascular disease prior to HD. Many patients with CKD have comorbid cerebrovascular diseases; however, this was included among the exclusion criteria in order to isolate the effect of HD on the cerebrovascular system. Another limitation of our study is the lack of comparison with a control group (CG) and other groups of patients with CKD in the early stages of the disease or undergoing peritoneal dialysis (PD) treatment. PD is a dialysis treatment that does not use an extracorporeal mechanism, such as HD, but an intracorporeal mechanism that is less stressful for the patient [34]. Such a comparison would have provided significant data and could have further defined the haemodynamic peculiarities of HD patients. Future studies should utilise a larger patient sample and compare cerebrovascular and cardiovascular indices, as well as cognitive performance, between the various stages of CKD and among patients receiving different treatments, including pharmacological, HD and PD.

## 5. Conclusions

Among HD patients, there is a high percentage of cognitive impairment (between 17% and 65%) associated with alterations of cerebral and carotid haemodynamics. In particular, statistically significant correlations emerged between cognitive performance and cerebral flow indices. Cerebral haemodynamics are thus a factor to be taken into consideration as a possible pathological mechanism underlying cognitive impairment in HD.

## Figures and Tables

**Table 1:** Sample features and HD data. Means, standard deviations and percentages of “age”, “gender”, “body mass index”, “body surface area” and HD data of the sample.

Total Patients	32
Age (mean ± SD)	65 ± 12
Gender (%)	Male 20 (62.5%) Female 12 (37.5%)
Body Mass Index (mean ± SD)	25 ± 6
Body Surface Area (mean ± SD)	2 ± 0.1
HD age	3 ± 4 years
Vascular access	● AVS (56.2%) ● CVC (40.36%) ● GRAFT (3.2%)
Filter type	● 1.4 (6.5%) ● 1.6 (15.6%) ● 1.7 (3.1%) ● 1.8 (65.6%) ● 2.1 (3.1%) ● 2.15 (6.2%) ● 2.2 (3.1%)
Type of HD	● HD standard (68.7%) ● AFB-K (15.6%) ● HDF-pre (9.38%) ● HDF-post (6.2%)
Sodium conductivity (mean)	141
Bicarbonate (mean)	32
Systolic blood pressure (mean)	132 mmHG
Diastolic blood pressure (mean)	71 mmHG
Mean arterial pressure	92.7 mmHG
Kt/v (mean ± SD)	1.30 ± 0.1
GFR mL/min (mean ± SD)	4 ± 1 mL/min/1.73 m^2^

**Table 2:** Risk factors. Means, standard deviations and percentages of risk factors in the sample.

Smoking	3.13%
Obesity	18.75%
Hypercholesterolaemia	28.13%
Hypertension	81.25%
Diabetes	53.12%
Atrial fibrillation	9.38%
Coronary heart disease	40.63%
Peripheral Arterial Obstructive Disease	18.75%
Stroke	9.38%
Iper-PTH	96.77%
C-reactive Protein	71.8%
Systemic Lupus Erythematosus	6.25%
Haemoglobin (media ± DS)	10.7 ± 0.5
Haematocrit (media ± DS)	35.8% ± 3
PH pre-HD (media ± DS)	73,155 ± 0.04
PH post-HD (media ± DS)	74,128 ± 0.04

**Table 3:** Cognitive data. Means, standard deviations and percentages of cognitive scores obtained in: (A) MoCa, (B) FAB and (C) TMT-A/B.

(A) Montreal Cognitive Assessment (MoCA)		
Sub-item	Total	Score (mean and DS)
Visuospatial and executive	5	2.4 ± 1.6
Naming	3	2.6 ± 0.6
Attention	6	4.4 ± 1.5
Language	3	1.6 ± 0.7
Abstraction	2	1.1 ± 0.7
Delayed recall	5	1.8 ± 1.7
Orientation	6	5.2 ± 0.7
Global results	19.3 ± 5.4	
Correct score	19.3 ± 4.5	
% of decline	17.24	
% of borderline	24.12	
% of normal	58.62	
(B) Frontal Assessment Battery (FAB)		
Sub-item	Total	Score (mean and DS)
Similarities	3	2.3 ± 0.7
Lexical fluency	3	2.2 ± 0.8
Motor series	3	1.9 ± 0.8
Conflicting instructions	3	2.4 ± 1
Go-no-go	3	1.8 ± 1.2
Prehension behaviour	3	2.3 ± 0.6
Global score	13.1 ± 3.8	
Correct score	13.2 ± 3.2	
% of deficit	44.83	
% of borderline	17.24	
% of normal	41.38	
% of higher mis	44.81	
(C) Trail-Making Test a-b		
Seconds	Score below norm	
110 s ± 75	55.17%	
234 s ± 148	65.52%	

**Table 4:** TCD data. Means, standard deviations and percentages of TCD data.

	Normal Values	Right (Mean)	Left (Mean)
PSV (cm/s)	≤160	74.2	72.3
EDV (cm/s)	51 ± 11	31.4	30
MFV (cm/s)	55 ± 12	45.2	44
RI	<0.8	0.57	0.56
PI	0.5–1.19	0.96	0.92
MFV test	60 ± 18	53.9	54.2
Seconds	25–30 s	27	26
BHI	1.03–1.65	0.69	0.82

**Table 5:** SAT data. Means, standard deviations and percentages of SAT data.

	Normal Values	Right (Mean or %)	Left (Mean or %)
IMT (mm)	<0.9	0.96	1.07
*n*° plaques	0	1.77	1.75
% of stenosis	0%	15.7%	15.4%
ICA PSV (cm/s)	<125	65.5	64.8
ICA EDV (cm/s)	<40	17.3	18.2
ICA CCA (cm/s)	<2	58.7	61
Stenosis degree			
<50%		15.30%	7.7%
50–60%		61.54%	92.3%
>70%		23.1%	0%

## Data Availability

The data are not publicly available due to privacy and ethical restrictions.

## Supplementary Materials

The following supporting information can be downloaded at: https://www.mdpi.com/article/10.3390/jcm14144890/s1, Table S1: Association between TCD and cognitive data; Table S2: Association between SAT and cognitive data; Table S3: Linear regression between cognitive scores and TCD; Table S4: Linear regression between cognitive scores and SAT.

## Abbreviations

The following abbreviations are used in this manuscript:

CKD	Chronic kidney disease
CI	Cognitive impairment
HD	Haemodialysis
SAT	Supra-Aortic Trunk Echodoppler
TCD	Transcranial Doppler
MoCA	Montreal Cognitive Assessment
FAB	Frontal Assessment Battery
TMT-a/b	Trail-Making Test a/b
CBF	Cerebral blood flow
CAD	Coronary artery disease
ESRD	End-stage renal disease
MFV	Mean cerebral flow velocity
PSV	Peak systolic velocity
EDV	End-diastolic velocity
PI	Gosling’s Pulsatility Index
RI	Pourcelot’s Resistance Index
BHI	Breath Hold Index
ICP	Intracranial pressure
MCA	Middle cerebral artery
IMT	Intima-media thickness
CCA	Common carotid artery
ICA	Internal carotid artery
BMI	Body Mass Index
BSA	Body Surface Area
CVR	Cerebrovascular reactivity
CG	Control group
PD	Peritoneal dialysis
