# Supplementary material for: Cerebral Haemodynamics and Cognitive Impairment in Chronic Haemodialysis Patients: A Pilot Study
Source: J Clin Med. 2025 Jul 10;14(14):4890. doi: 10.3390/jcm14144890 (PMC12295371; doi:10.3390/jcm14144890)
Supplement: Supplementary file 1 [file jcm-14-04890-s001.zip › jcm-3685551-supplementary.pdf]

Supplemental materials

Table S1: Association between TCD and Cognitive data The table shows T-tests between TCD and cognitive scores: degrees of freedom, Student's t(s), and p-values are indicated. Statistical significance was marked (** p < .012, *** p < .001).

S1. TCD AND COGNITIVE DATA		T-test	dof	p
PSV left	MoCA	12.34	19.0	<.001***
	FAB	14.67	19.0	<.001***
	TMT-a	-1.72	19.0	0.102
	TMT-b	-5.76	19.0	<.001***
EDV left	MoCA	6.33	19.0	<.001***
	FAB	11.73	19.0	<.001***
	TMT-a	-4.64	19.0	<.001***
	TMT-b	-7.96	19.0	<.001***
MFV left	MoCA	10.60	19.0	<.001***
	FAB	15.20	19.0	<.001***
	TMT-a	-3.61	19.0	0.002**
	TMT-b	-7.19	19.0	<.001***
RI left	MoCA	-20.62	19.0	<.001***
	FAB	-16.83	19.0	<.001***
	TMT-a	-6.66	19.0	<.001***
	TMT-b	-9.36	19.0	<.001***
PI left	MoCA	-20.31	19.0	<.001***
	FAB	-16.45	19.0	<.001***
	TMT-a	-6.63	19.0	<.001***
	TMT-b	-9.34	19.0	<.001***
MFV TEST left	MoCA	12.53	19.0	<.001***
	FAB	16.84	19.0	<.001***
	TMT-a	-2.90	19.0	0.009**
	TMT-b	-6.70	19.0	<.001***
BHI left	MoCA	-20.45	19.0	<.001***
	FAB	-16.80	19.0	<.001***
	TMT-a	-6.63	19.0	<.001***

	TMT-b	-9.35	19.0	<.001***
PSV right	MoCA	13.42	22.0	<.001***
	FAB	15.43	22.0	<.001***
	TMT-a	-1.38	22.0	0.180
	TMT-b	-6.01	22.0	<.001***
EDV right	MoCA	7.31	22.0	<.001***
	FAB	11.94	22.0	<.001***
	TMT-a	-5.02	22.0	<.001***
	TMT-b	-8.11	22.0	<.001***
MFV right	MoCA	12.19	22.0	<.001***
	FAB	16.18	22.0	<.001***
	TMT-a	-3.78	22.0	0.001**
	TMT-b	-7.40	22.0	<.001***
RI right	MoCA	-22.78	22.0	<.001***
	FAB	-29.57	22.0	<.001***
	TMT-a	-8.08	22.0	<.001***
	TMT-b	-9.76	22.0	<.001***
PI right	MoCA	-22.06	22.0	<.001***
	FAB	-28.65	22.0	<.001***
	TMT-a	-8.05	22.0	<.001***
	TMT-b	-9.74	22.0	<.001***
MFV TEST right	MoCA	12.54	22.0	<.001***
	FAB	15.57	22.0	<.001***
	TMT-a	-2.99	22.0	0.007**
	TMT-b	-6.93	22.0	<.001***
BHI right	MoCA	-22.96	22.0	<.001***
	FAB	-31.32	22.0	<.001***
	TMT-a	-8.05	22.0	<.001***
	TMT-b	-9.74	22.0	<.001***

Table S2: Association between SAT and Cognitive data. Table shows T-tests between SAT and cognitive scores: degrees of freedom, Student's t and p-values are indicated. Statistical significance was marked (** p ≤ .012, *** p < .001).

S2. SAT and COGNITIVE DATA		T-test	dof	p
**IMT right**	**MoCA**	-26.246	12.0	<.001***
	**FAB**	-24.307	12.0	<.001***
	**TMT-a**	-5.861	12.0	<.001***
	**TMT-b**	-6.040	12.0	<.001***
**Plaques right**	**MoCA**	-25.515	12.0	<.001***
	**FAB**	-20.310	12.0	<.001***
	**TMT-a**	-5.801	12.0	<.001***
	**TMT-b**	-6.015	12.0	<.001***
**Stenosis right**	**MoCA**	-1.126	12.0	0.282
	**FAB**	0.380	12.0	0.711
	**TMT-a**	-4.563	12.0	<.001***
	**TMT-b**	-5.454	12.0	<.001***
**PSV ICA right**	**MoCA**	7.507	12.0	<.001***
	**FAB**	8.504	12.0	<.001***
	**TMT-a**	-1.836	12.0	0.091
	**TMT-b**	-3.899	12.0	0.002**
**EDV ICA right**	**MoCA**	-0.927	12.0	0.372
	**FAB**	1.363	12.0	0.198
	**TMT-a**	-4.751	12.0	<.001***
	**TMT-b**	-5.504	12.0	<.001***
**PSV CCA right**	**MoCA**	6.911	12.0	<.001***
	**FAB**	7.709	12.0	<.001***

	**TMT-a**	-2.158	12.0	0.052
	**TMT-b**	-4.068	12.0	0.002**
**Stenosis DOPPLER right**	**MoCA**	4.844	12.0	<.001***
	**FAB**	5.687	12.0	<.001***
	**TMT-a**	-3.261	12.0	0.007**
	**TMT-b**	-4.700	12.0	<.001***
**IMT left**	**MoCA**	-27.356	12.0	<.001***
	**FAB**	-24.897	12.0	<.001***
	**TMT-a**	-5.849	12.0	<.001***
	**TMT-b**	-6.036	12.0	<.001***
**Plaques left**	**MoCA**	-23.298	12.0	<.001***
	**FAB**	-17.374	12.0	<.001***
	**TMT-a**	-5.829	12.0	<.001***
	**TMT-b**	-6.029	12.0	<.001***
**Stenosis left**	**MoCA**	-0.960	12.0	0.356
	**FAB**	0.247	12.0	0.809
	**TMT-a**	-5.297	12.0	<.001***
	**TMT-b**	-5.927	12.0	<.001***
**PSV ICA left**	**MoCA**	6.577	12.0	<.001***
	**FAB**	7.247	12.0	<.001***
	**TMT-a**	-2.230	12.0	0.046
	**TMT-b**	-4.215	12.0	0.001**
**EDV ICA left**	**MoCA**	-0.454	12.0	0.658
	**FAB**	1.403	12.0	0.186
	**TMT-a**	-4.712	12.0	<.001***
	**TMT-b**	-5.449	12.0	<.001***

**PSV CCA left**	**MoCA**	8.927	12.0	<.001***
	**FAB**	10.239	12.0	<.001***
	**TMT-a**	-2.144	12.0	0.053
	**TMT-b**	-4.099	12.0	0.001**
**Stenosis DOPPLER left**	**MoCA**	7.368	12.0	<.001***
	**FAB**	8.510	12.0	<.001***
	**TMT-a**	-2.708	12.0	0.019
	**TMT-b**	-4.443	12.0	<.001***

Tabel S3. Linear regression between cognitive scores and TCD
The table shows linear regressions between a) MoCa and TCD right; b) MoCA and TCD left; c)FAB and TCD right; d) FAB and TCD left; e) TMT-a and TCD right; f) TMT-a and TCD left; g) TMT-b and TCD right; h) TMT-b and TCD left. Statistical significance was marked (** p ≤ .012, *** p < .001).

**a) MoCA- TCD RIGHT**			**R**	**R^2**	**b) MoCA- TCD LEFT**		**R**	**R^2**
**Model 1**			**0.365**	**0.133**	Model 2		**0.501**	**0.251**
Predictor	Estimate	SE	t	p	Estimate	SE	t	p
Intercept	12.4084	16.081	0.7716	0.452	11.855	25.874	0.4582	0.655
PSV	-0.0580	0.379	-0.1528	0.881	-0.242	1.035	-0.2338	0.819
EDV	-0.0384	0.489	-0.0785	0.939	-0.907	1.650	-0.5496	0.593
MFV	0.1652	0.733	0.2254	0.825	0.507	0.971	0.5216	0.611
RI	29.0646	55.553	0.5232	0.608	49.763	138.198	0.3601	0.725
PI	-12.8710	20.103	-0.6402	0.532	-17.455	107.597	-0.1622	0.874
MFV TEST	-0.0145	0.507	-0.0287	0.977	0.323	0.573	0.5629	0.584
BHI	2.0383	6.759	0.3016	0.767	-0.300	7.679	-0.0391	0.969
**c) FAB – TCD RIGHT**			**R**	**R^2**	**d) FAB – TCD LEFT**		**R**	**R^2**
**Model 3**			**0.625**	**0.391**	**Model 4**		**0.681**	**0.464**
Intercept	-0.8947	7.234	-0.1237	0.903	37.584	18.179	2.0674	0.061
PSV	-0.0716	0.171	-0.4196	0.681	0.947	0.727	1.3019	0.217
EDV	0.1070	0.220	0.4860	0.634	-2.482	1.159	-2.1414	0.053
MFV	0.0812	0.330	0.2462	0.809	-0.431	0.682	-0.6310	0.540
RI	31.7571	24.992	1.2707	0.223	172.962	97.097	1.7813	0.100

PI	-7.7638	9.044	-0.8584	0.404	-135.329	75.596	-1.7902	0.099
MFV TEST	0.0123	0.228	0.0541	0.958	0.527	0.403	1.3079	0.215
BHI	1.8059	3.041	0.5939	0.561	-0.527	5.395	-0.0977	0.924
**e) TMT-A - TCD RIGHT**			**R**	**R^2**	**f) TMT-A- TCD LEFT**		**R**	**R^2**
**Model 5**			**0.776**	**0.602**	**Model 6**		**0.680**	**0.462**
Intercept	367.13	152.55	2.407	0.029	273.49	380.22	0.719	0.486
PSV	3.17	3.60	0.882	0.392	2.51	15.22	0.165	0.872
EDV	1.01	4.64	0.217	0.831	28.98	24.25	1.195	0.255
MFV	-15.93	6.95	-2.292	0.037	-11.53	14.27	-0.808	0.435
RI	-290.36	527.02	-0.551	0.590	-1884.38	2030.81	-0.928	0.372
PI	77.27	190.72	0.405	0.691	976.48	1581.12	0.618	0.548
MFV TEST	6.64	4.81	1.382	0.187	-11.23	8.43	-1.332	0.208
BHI	-127.68	64.12	-1.991	0.065	60.95	112.84	0.540	0.599
g) TMT-B- TCD **RIGHT**			**R**	**R^2**	h) TMT-B – TCD RIGHT		**R**	**R^2**
**Model 7**			**0.694**	**0.481**	**Model 8**		**0.612**	**0.375**
Intercept	301.55	344.47	0.875	0.395	440.44	629.0	0.7002	0.497
PSV	3.54	8.13	0.436	0.669	1.55	25.2	0.0616	0.952
EDV	6.76	10.48	0.645	0.529	37.75	40.1	0.9411	0.365
MFV	-30.33	15.70	-1.933	0.072	-18.93	23.6	-0.8018	0.438
RI	999.19	1190.02	0.840	0.414	-2443.57	3359.8	-0.7273	0.481
PI	-276.42	430.64	-0.642	0.531	1363.82	2615.8	0.5214	0.612
MFV TEST	12.41	10.85	1.143	0.271	-10.59	13.9	-0.7600	0.462
BHI	-226.49	144.78	-1.564	0.139	65.49	186.7	0.3508	0.732

Table S4. Linear regression between cognitive scores and SAT

The table shows linear regressions between a) MoCa and SAT right; b) MoCA and SAT left; c)FAB and SAT right; d) FAB and SAT left; e) TMT-a and SAT right; f) TMT-a and SAT left; g) TMT-b and SAT right; h) TMT-b and SAT left. Statistical significance was marked (** p ≤ .012, *** p < .001).

Model Cognitive data – SAT

**a) MOCA-SAT RIGHT**	**R**	**R^2**	**b) MOCA-SAT LEFT**	**R**	**R^2**
**Model 1**	**0.820**	**0.673**	Model 2	**0.591**	**0.349**

Model Cognitive data – SAT

**Predictor**	**Estimate**	**SE**	**t**	p	Estimate	SE	t	p
Intercept	21.8508	3.3681	**6.488**	0.001	16.68118	6.9084	2.4146	0.061
IMT	-4.7880	3.5490	-1.349	0.235	5.46285	6.8718	0.7950	0.463
Plaques	0.3108	0.7509	0.414	0.696	0.19158	1.4424	0.1328	0.900
Stenosis	-0.0191	0.0677	-0.282	0.789	-0.04572	0.1019	-0.4486	0.672
PSV ICA	0.0479	0.0477	1.003	0.362	-0.09990	0.0926	-1.0783	0.330
EDV ICA	-0.2096	0.1483	-1.413	0.217	0.17125	0.1877	0.9126	0.403
PSV CCA	0.0921	0.0450	2.048	0.096	0.00540	0.1120	0.0482	0.963
Stenosis DOPPLER	-0.0590	0.0463	-1.273	0.259	0.00619	0.0744	0.0832	0.937
**c) FAB- SAT RIGHT**			**R**	**R^2**	**d) FAB- SAT LEFT**		**R**	**R^2**
**Model 3**			**0.935**	**0.875**	**Model 4**		**0.612**	**0.375**
Intercept	15.6350	1.6024	9.757	<.001	15.30204	6.8856	2.2223	0.077
IMT	-3.5552	1.6885	-2.106	0.089	-0.42725	5.5423	-0.0771	0.942
Plaques	-0.2539	0.3572	-0.711	0.509	0.33831	1.0537	0.3211	0.761
Stenosis	0.0143	0.0322	0.444	0.675	-0.00543	0.0664	-0.0817	0.938
PSV ICA	0.0708	0.0227	3.117	0.026	-0.06260	0.0504	-1.2433	0.269
EDV ICA	-0.2090	0.0706	-2.962	0.031	0.06296	0.1191	0.5284	0.620
PSV CCA	0.0628	0.0214	2.932	0.033	0.05859	0.0736	0.7962	0.462
Stenosis DOPPLER	-0.0452	0.0220	-2.051	0.096	-0.02883	0.0456	-0.6324	0.555
**e) TMT-A- SAT RIGHT**			**R**	**R^2**	**f) TMT-A – SAT LEFT**		**R**	**R^2**
**Model 5**			**0.914**	**0.836**	**Model 6**		**0.813**	**0.660**
Intercept	109.996	60.901	1.806	0.131	124.909	168.63	0.741	0.492
IMT	80.949	64.172	1.261	0.263	22.613	135.73	0.167	0.874
Plaques	-11.305	13.577	-0.833	0.443	-31.439	25.81	-1.218	0.277
Stenosis	0.529	1.223	0.432	0.683	1.806	1.63	1.110	0.317
PSV ICA	-2.267	0.863	-2.626	0.047	3.131	1.23	2.539	0.052
EDV ICA	3.095	2.682	1.154	0.301	-4.683	2.92	-1.605	0.169
PSV CCA	-1.818	0.814	-2.235	0.076	-2.532	1.80	-1.405	0.219
Stenosis DOPPLER	2.595	0.838	3.098	0.027	0.345	1.12	0.309	0.770
**g) TMT-B – SAT RIGHT**			**R**	**R^2**	**h) TMT-B – SAT LEFT**		**R**	**R^2**
**Model 7**			**0.765**	**0.585**	**Model 8**		**0.813**	**0.661**

Model Cognitive data – SAT

Intercept	376.78	198.34	1.8997	0.116	559.22	345.29	1.620	0.166
IMT	-15.60	208.99	-0.0746	0.943	-220.34	277.92	-0.793	0.464
Plaques	6.31	44.22	0.1427	0.892	-28.27	52.84	-0.535	0.616
Stenosis	1.07	3.98	0.2679	0.799	4.20	3.33	1.262	0.263
PSV ICA	-4.82	2.81	-1.7162	0.147	5.46	2.52	2.163	0.083
EDV ICA	8.47	8.73	0.9702	0.377	-9.09	5.97	-1.521	0.189
PSV CCA	-3.56	2.65	-1.3436	0.237	-3.51	3.69	-0.952	0.385
Stenosis DOPPLER	4.17	2.73	1.5272	0.187	-1.69	2.29	-0.738	0.494
